# Influence of climatic factors on cyanobacteria and green algae development on building surface

**DOI:** 10.1371/journal.pone.0282140

**Published:** 2023-03-06

**Authors:** Paloma Reboah, Clarisse Balland Bolou-Bi, Sophie Nowak, Aurélie Verney-Carron

**Affiliations:** 1 LEESU, Univ Paris Est Creteil, Ecole de ponts, Creteil, France; 2 Univ Paris Est Creteil and Université Paris Cité, CNRS, LISA, Créteil, France; 3 Université Paris Cité, CNRS, ITODYS, Paris, France; 4 Université Paris Cité and Univ Paris Est Créteil, CNRS, LISA, Paris, France; King Abdulaziz University, SAUDI ARABIA

## Abstract

Buildings and monuments are often colonized by microorganisms that can result in colour change and aesthetical and physico-chemical damages. This bio-colonization is dependent of the material and on the environment. In order to better understand and correlate the microbial development at the surface of buildings with meteorological parameters, concentration of green algae and cyanobacteria have been measured using an *in situ* instrument on the wall of a private habitation in the Parisian region during two periods: spring and fall-winter. Different locations were also chosen to assess the influence of the position (horizontal or vertical) and of the situation (shaded vs. sunny microclimate). The results show that the microorganism development rapidly responds to rainfall events but the response is more intense in winter as temperature is lower and relative humidity (RH) higher. Cyanobacteria are less sensitive to this seasonal effect as they are more resistant to desiccation than green algae. Based on all the data, different dose-response functions have been established to correlate RH, rain and temperature to the green algae concentration. The influence of the microclimate is considered via specific fitting parameters. This approach has to be extended to new campaign measurements but could be very useful to anticipate the effect of climate change.

## Introduction

Building materials are affected by different biological and chemical processes that can induce their deterioration. Especially, different microorganisms are able to colonize building materials, like green algae and cyanobacteria in biofilm forms, bacteria, moss, lichen and fungi [1]. Cyanobacteria and algal presence is largely documented on monument surfaces [2–5] and are considered as first colonizers of stone monuments [6–8]. These microorganisms can induce discolorations (dark, green or reddish) on the building surfaces [9]. For example, cyanobacteria and green algae are frequently visible by the presence of colored biofilm development on building façades [10]. Microorganisms can also deteriorate monuments by biological, physical and chemical actions. Lichen and moss can weaken physically the substrate by hyphae and rhizoids extension in porous network [11]. Microorganisms are attached to the substrate by biofilm formation and its properties of water retention can induce mass expansion due to the volume change and deterioration [12]. A biofilm also increases long-term substrata moisture and can induce a breakdown of the pore network during freezing periods [13, 14]. In addition, cyanobacteria and algae secrete organic acids that can dissolve the minerals. They can also generate organic matter for heterotroph microorganisms and favor their growth [14].

Biological colonization on façade is controlled by extrinsic (environment) and intrinsic factors (building materials) [10]. The main environmental factors are: water availability, temperature, relative humidity and insolation. Water availability is the most important factor for all microorganisms development [2] and light is the key factor for phototrophic microorganisms growth. Algal communities that are associated to high humidity and water retention [12]. In the same way, sufficient water availability combined to medium temperature allow the growth of variety of cyanobacteria, despite to their great resistance to desiccation [7]. Colonization by phototrophic microorganisms, especially cyanobacteria, was correlated with air humidity and light intensity [15]. Other environmental factors, such as temperature, pH or light intensity influence the distribution of microorganisms [12].

Concerning intrinsic factors, biogeochemical deterioration processes are controlled by the chemistry of the substrate and biogeophysical deterioration processes by substrate properties, such as porosity and roughness [2, 16]. However, more and more modern buildings or monuments are covered with plaster on the façade and surrounded by composite materials on the floor, which also undergo deterioration with aesthetically unsightly colorations. Unfortunately, unlike natural stones, these damages are much less documented in the literature. However, even if chemical composition and physical properties (porosity, roughness) of coating or slab are different, they present similarities. For example, plaster is generally composed by a mix of gypsum powder, calcic or dolomitic chalk, sand and water, which is sprayed on walls. Thus, plaster is a cementitious material and has a capillary-porous structure, which can be responsible for many unfavorable phenomena, such as excessive dampness of the material, capillary rise, microbial colonization or crystallization of salts on material surfaces. The objective of this work is therefore to study the biological colonization of materials such as plaster-rendered façade and composite tiles, used for modern housing.

From a methodological point of view, most of the works dealing with microbial colonization used traditional techniques from cultivation in a rich medium until DNA extraction and amplified ribosomal DNA restriction analysis targeting the 16S rRNA genes. These techniques are effective in analyzing the presence of microorganisms at a given time, but unfortunately do not give an indication of their dynamics over time according to climatic factors. Especially, it is very important to know the key parameters controlling their presence and dynamics. Since several years, new non-destructive and fast techniques have been developed to measure the *in situ* concentration of microorganisms. The BenthoTorch (bbe Moldaenke ®) is one of this *in situ* instrument allowing a fast benthic microorganism (green algae, cyanobacteria and diatoms) concentration measurement by chlorophyll a fluorescence. This instrument is generally used in river, freshwater or in caves, and recently on ceramic tiles [17].

The objective of our study is to improve our knowledge about the climatic influence on microbial development and their dynamic over time in order to understand the impact of climate change on plaster and tiles deteriorations under temperate climate. For that, two different contrasted seasons were chosen: spring and fall winter.

## Materials and methods

### Site and measurement zones

The studied site is a private habitation located in the Parisian suburb (4°5’0” N; 2°47’24” E). Access was permitted by the homeowner. In the Parisian region, the climate is described as hot temperate (Cf. Koppen and Geiger classification) with the presence of rainfall in driest months. The annual average temperature is 11.6°C and annual average rainfall is 693.6 mm for 1981–2010 period (infoclimat.fr).

Two campaigns of measurement were carried out: the first one between May 7th 2020 and June 18th 2020 (spring) and the second campaign between November 5th 2020 and December 30th 2020 (fall-winter). During both campaigns, climatic data (daily mean relative humidity (%), daily mean temperature (°C) and precipitation (mm)) were collected from the Roissy-en-France meteorological station (infoclimat.fr).

**Fig 1:**
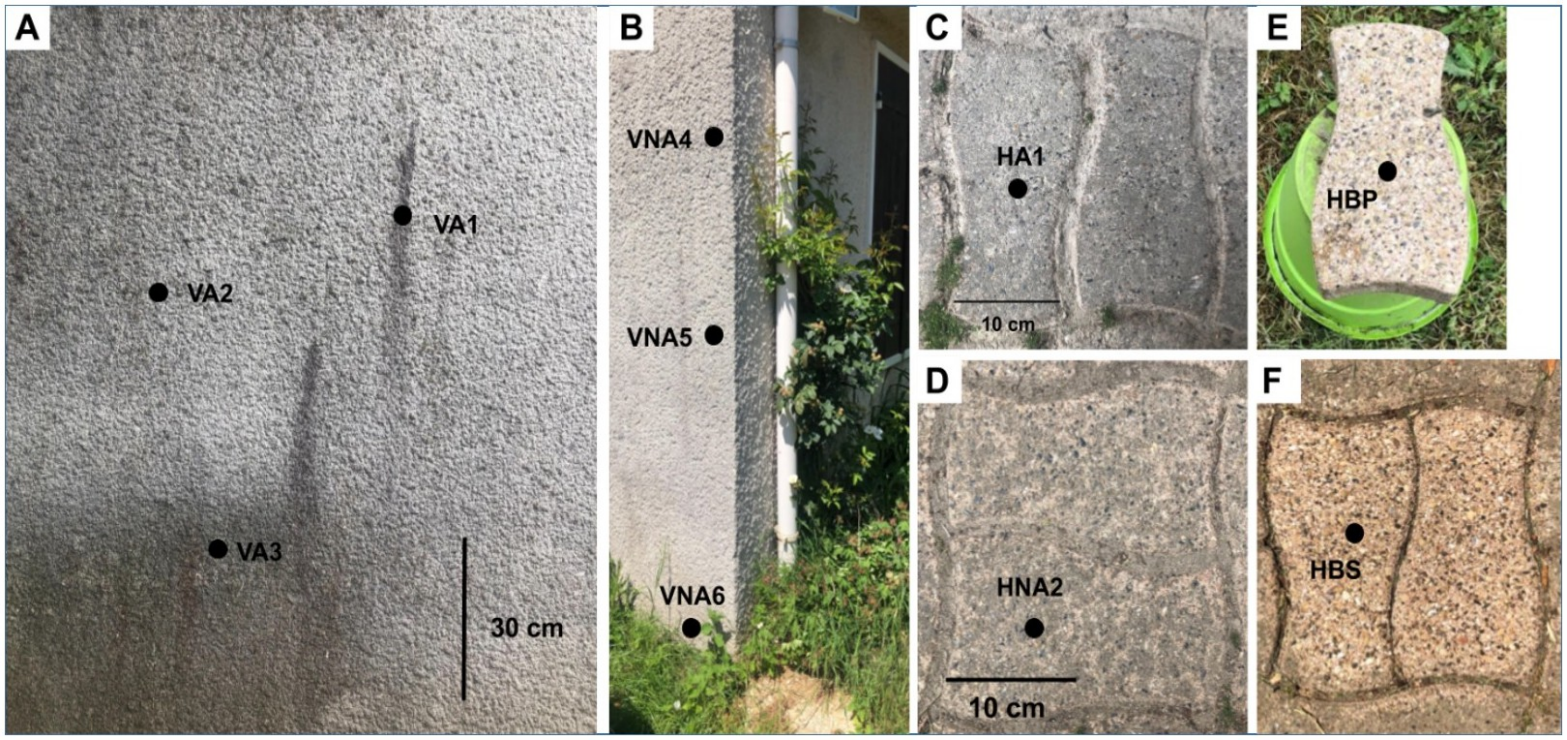
Spring campaign measurement zones. Vertical positions sheltered by tree (A) and not sheltered (B), horizontal positions sheltered by tree (C), not sheltered (D), horizontal bleach-washed tiles (E & F). E is not in contact with other tiles and soil contrary to F.

Different zones of measurements were selected (Fig 1) to assess the role of different parameters: the position, the orientation and the sheltered by a tree or not situation. Vertical zones (wall surfaces) are made out roughcast that is a mix between coarse sand (millimetric clasts) and concrete (Fig 1A and 1B). The horizontally surfaces are composite tiles composed by concrete, pebble clasts (millimetric to centimetric clast size) and bioclasts like gastropods (Fig 1C and 1D). Even if the nature of materials is different, the comparison is interesting to assess the role of the position. Indeed, horizontal position can potentially retain more water than vertical position, which influences the microbial diversity [18].

For the vertical surface, two areas were selected: one close to a chestnut (sheltered situation), that limits the direct impact of rainfall (VA with 3 measurement zones, VA1-2-3, at 100, 143 and 182 cm high, respectively, Fig 1A) and favors a high humidity and one not sheltered by the tree (VNA with also 3 zones, VNA4-5-6, at 200, 150 and 15 cm high, respectively, Fig 1B). Vertical surfaces display some colour changes and weathering forms. On VA1, there are reddish runs compared to VA2. VA3 is located in a humid area due to the proximity of a waste bin. The non-sheltered vertical surfaces display less color change. VA2 did not show significant color change, unlike VA1 and VA3 with reddish and greyish coloration, respectively. VA position are located on north oriented facades and VNA on south oriented façade.

For the horizontal position, two zones were also chosen, sheltered (HA1) and not sheltered (HNA2) by the tree. Moreover, in order to understand biological colonization behavior, two tiles were cleaned with bleach: one on the soil (HBS, Fig 1F) and one raised on a bucket to avoid lateral contamination (HBP Fig 1E). Measurements on HBP (Fig 1E) was realized only during spring season.

### Characterization of the materials: Plaster and tiles

The studied wall surface is made out of roughcast that is a mix between coarse sand (millimetric clasts) and concrete (Fig 1A and 1B). Presence of CaCO_3_ on surface was determined by HCl test. The mineralogy was determined by X-Ray Diffraction (XRD) using a Panalytical Empyrean powder diffractometer equipped with a PIXcel detector fitted with a Cu anode tube (Kα1 = 1.5406 Å) operating at 45 kV and 40 Ma. The crystalline phases were identifying and quantifying with HighScorePlus using ISCD and COD database.

### Biological analyses

Microorganism concentrations (green algae and cyanobacteria) were daily measured on each zone using a BenthoTorch (bbe Moldaenke®). This portable field instrument consists of *in situ* measurement of benthic microorganisms concentration (green algae and cyanobacteria in μg chl-a/cm^2^) using *in vivo* fluorescence of chlorophyll on various substrates such as stones and sediments. The BenthoTorch directly calculates the biomass based on the chlorophyll-a content and determines the distribution over the different categories of algae. All measurements were conducted in triplicate and realized each day evening (~9 pm).

### DNA extraction and NGS of 16S and ITS

As significant differences have been observed between vertical spots for spring and winter seasons, the sequencing was focused on these vertical positions. Sampling with sterile swab stick has been realized on each point of BenthoTorch measurement on July 23, 2021. Total genomic DNA was extracted from sampling swab with Fast DNA Spinkit for Soil (MPBio®) according to manufacturer’s instructions. DNA extract was amplified and sequenced on Eurofins genomics platform for 16S on V3-V5 region for bacteria and ITS on ITS1 region for fungi. Green algae were not sequenced. Sequencing data analyses were performed on FROGS pipeline. FROGS allow to obtain abundance table of operational taxonomic unit (OTU) and their taxonomic affiliations. Databases used for taxonomic affiliation are Silva 138 for 16S and UNITE 8.0 for ITS. Alpha-diversity and Beta-diversity indices were calculated using Easy 16S, Migale platform to observe the richness of each sample and the phylogenic difference between the samples, respectively.

### Data treatment

Statistical analyses were carried out using Rstudio ® for the Pearson correlation matrices. Data have been fitted using Matlab®.

## Results

### Mineralogical composition of materials

**Fig 2:**
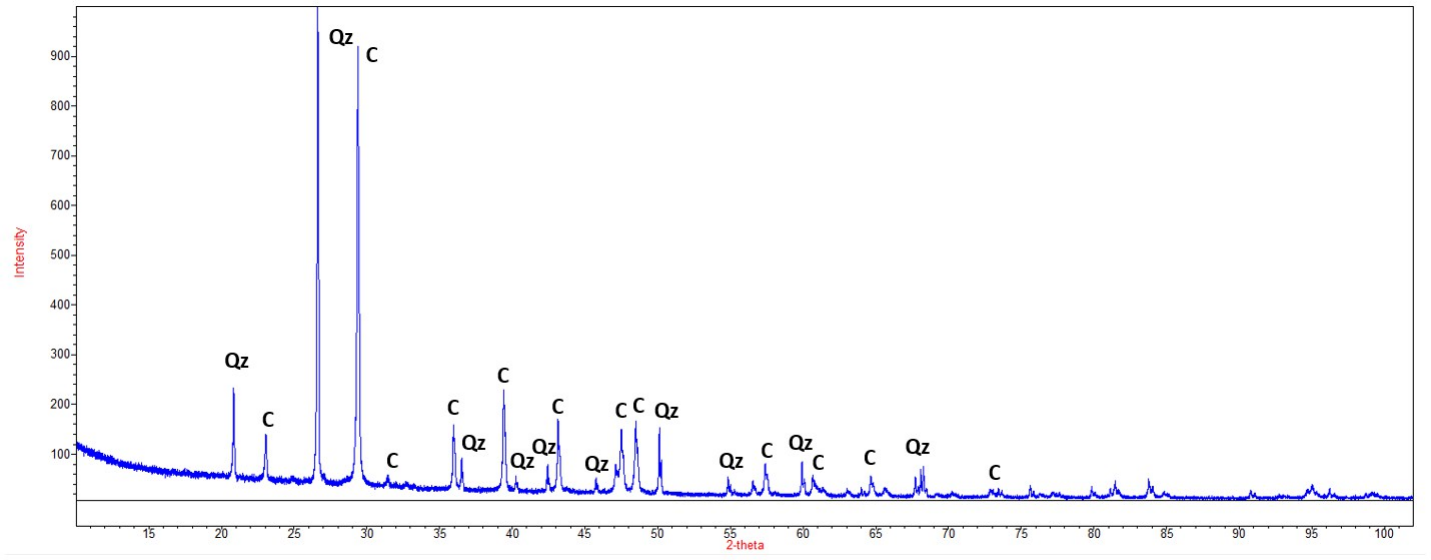
XRD analysis of the wall vertical surfaces. C is for calcite (00-900-9666) and Qz for quartz (00-900-0095) from COD 1906 database.

Wall surfaces are covered by a plaster roughcast that is a mix between coarse sand (millimetric clasts) and concrete (Fig 1A and 1B). XRD analyses on the surfaces indicate that the plaster is mainly composed by 45% of calcite and 55% of quartz (Fig 2). The horizontally surfaces are composite tiles composed by concrete, pebble clasts (millimetric to centimetric clast size) and bioclasts like gastropods (Fig 1C and 1D).

### Climatic data

The climatic data are presented in Table 1. The total precipitation quantity is 57.8 mm for the spring period (from May 7th 2020 to June 18th 2020) and 133.4 mm for the fall-winter period (from November 5th 2020 to December 30th 2020). The average temperature is 16.4°C during the spring period and 7.9°C during the fall-winter one. During the studied spring season, daily temperature varies between 4.4°C and 15.9°C and during fall-winter season, between 2.1°C and 9.7°C. Relative humidity (RH) data also show significant differences for both periods. The percentage of time corresponding to the 3 categories RH < 40% (dry air), 40 < RH < 80% (medium air humidity), and RH > 80% (wet air) was calculated. During spring season, the major part of time (65%) air has a RH between 40% and 80%. Dry air period represents 15% of time and 20% of time for wet air. In the fall-winter season, no dry air (RH< 40%) was recorded. Air is considered wet with 78% of time of HR superior to 80%. In summary, the fall-winter period was cold, humid and rainy and the spring period was relatively hot and dry.

**Table 1:** Climatic data.

	Spring	Fall-winter
Monitoring period	05/07/2020–06/18/2020	11/05/2020–12/30/2022
Total precipitation (mm)	57.8	133.4
Average temperature (°C)	16.4	7.9
Temperature min-max	4.4–15.9	2.1–9.7
RH dry (<40%)	15	-
RH medium (40%<RH<80%)	65	22
RH wet (<80%)	20	78

### Concentrations in cyanobacteria and green algae

#### Spring period

**Fig 3:**
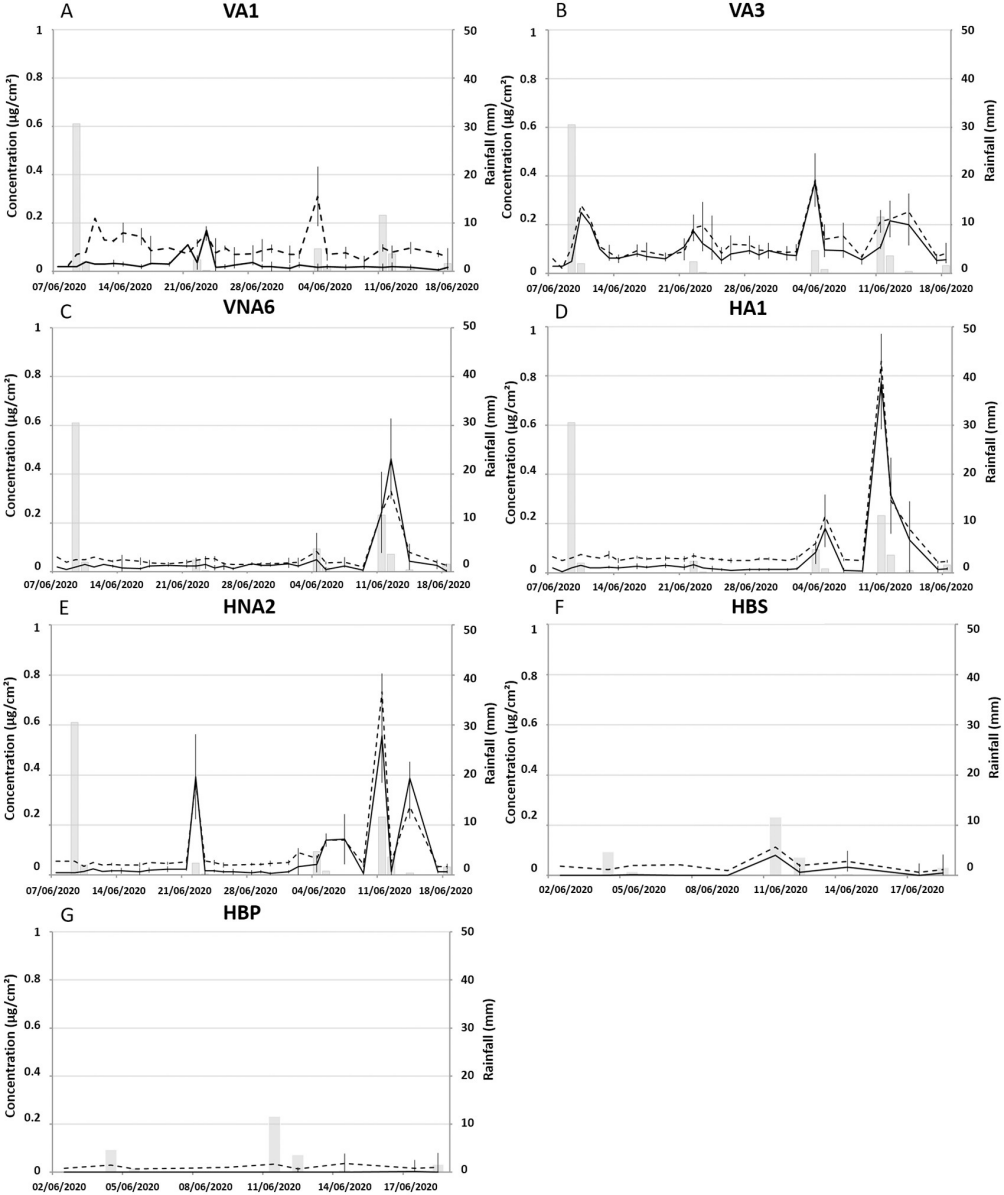
Evolution of microbial concentration (cyanobacteria and green algae) associated with rainfall event for the spring season campaign: For vertical (VA1 (A), VA3 (B), VNA6 (C)), horizontal positions (HA1 (D) and HNA2 (E)) and bleach washed tiles (HBS (F) and HBP (G)). Each point and bar represent the mean and standard deviation based on three replicates.

For VA2, VNA4 and VNA5, the concentrations remained always lower than limit of detection of the apparatus (0.02 μg/cm^2^, S1 Table). In the sheltered zones, for VA1 and VA3 (Fig 3A and 3B), the concentrations of green algae and cyanobacteria varied between 0.02 and 0.38 μg/cm^2^ and the concentrations in cyanobacteria were systematically higher than green algae. Rainy events are followed by a strong increase in cyanobacteria concentration for VA1, contrary to green algae concentration that seems to be less sensitive. For VA3, cyanobacteria and green algae displayed the same concentrations and have the same behavior with a proliferation more important during a rainy event.

In the unsheltered zone (Fig 3C), targeted microorganism concentrations remained low except after the 06/11 rainfall event, where a peak is observed for VNA6 (located near soil and plants (~10 cm)) that reached 0.30 μg/cm^2^ for cyanobacteria and 0.42 μg/cm^2^ for green algae. On the other hand, there is no increase of microorganism concentrations after the 05/09 rain event due to the drying before the measurement. However, measurements were made at 9pm and the rain event took place between 11pm and 1am.

For HA1 and HNA2 (Fig 3D and 3E), cyanobacteria and green algae had the same concentration and the same behavior. Horizontal positions display similar results to VNA6 (Fig 3C) with an increase of microbe concentrations during rainy event until 0.82 μg/cm^2^ for HA1 and 0.70 μg/cm^2^ for HNA2. The absence of peak is also observed after the rainy event of 05/09 due to the drying before the measurement. Indeed, one of our hypothesis was that during a rain event, the increase of concentration observed was due to a contamination of the paving stones placed right next to each other. Initially, the concentration was lower than the detection limit indicating that algae were completely killed after washing. For HBP (Fig 3G), concentrations remain low or null. For HBS (Fig 3F), the concentrations are under the detection limit for green algae and around 0.02 μg/cm^2^ for cyanobacteria until the 11/06/2020, where the concentration increases until ~ 0.1 μg/cm^2^ for both types of algae just after a rain event, which confirms our hypothesis.

#### Fall winter period

**Fig 4:**
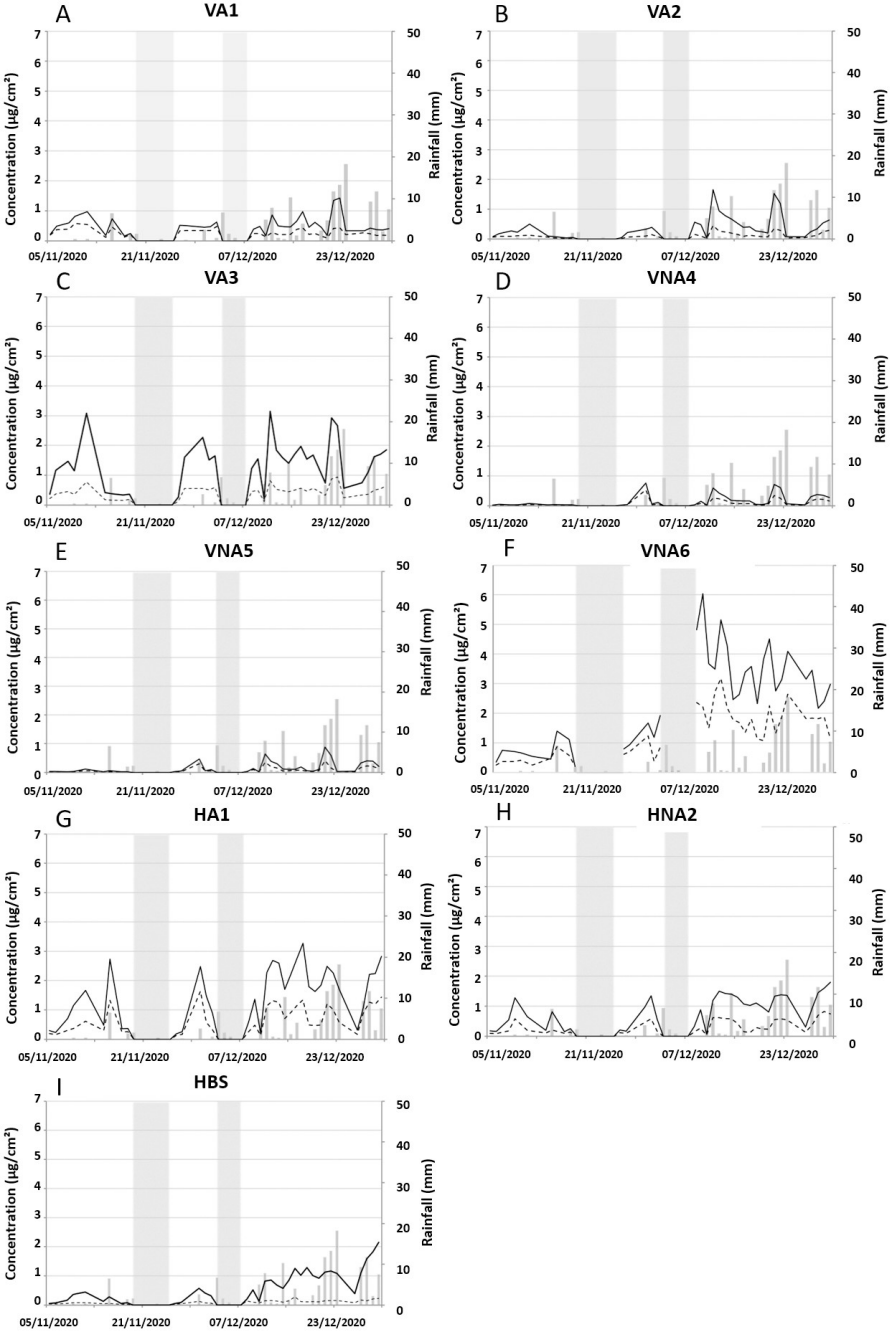
Evolution of microbial concentration (cyanobacteria and green algae) associated with rainfall event in the fall-winter season. For vertical sheltered (VA1 (A), VA2 (B), VA3 (C)), vertical non-sheltered (VNA4 (D), VNA5 (E), VNA6 (F)), horizontal positions (HA1 (G) and HNA2 (H)) and bleach washed tiles (HBS (I)). Each point and bar represent the mean and standard deviation based on three replicates. Grey zones correspond to the absence of measurements.

During this period, the influence of the tree and its sheltering effect was less relevant than in spring (Fig 4). For vertical positions (VA and VNA) (Fig 4A–4F), the concentration evolution was similar for the 6 zones. However, variations are more pronounced for VA3 (like in spring) (Fig 4C) and especially for VNA6 (Fig 4F), were lichen formation is visible close to the soil (and vegetation) and sensitive to capillary rise (Fig 1B). For horizontal positions (Fig 4G and 4H), algae concentrations for HA1 were slightly higher than HNA2 and reached 1.93 μg/cm^2^ for HA1 and 1.1 μg/cm^2^ for HNA2. This can be explained by some preferential runoffs in the HA1 area. Whatever the position, the concentration of green algae and cyanobacteria are 5 to 50 times more important in winter that in spring and reached 6 μg/cm^2^.

**Fig 5:**
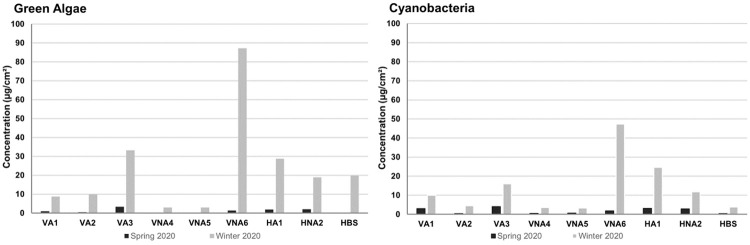
Cumulative microorganism concentrations (green algae and cyanobacteria) for different measurement areas and seasons: Spring and fall-winter. (Sum of each concentration by day of measurement). HBP is not presented because measurements were realized only during the spring season.

Microorganism cumulative concentrations (Fig 5) showed an important concentration in winter, up to ~47.3 μg/cm^2^ for cyanobacteria and 87.1μg/cm^2^ for green algae, than in summer with a maximum of 4.1 μg/cm^2^ for cyanobacteria and 3.3 μg/cm^2^ for green algae. The highest concentrations of microorganisms are observed at the end of the campaign during the rainiest period. Whatever the position, green algae concentrations were always higher than cyanobacteria.

### Microbial community on building surfaces

The investigation on microbial communities were carried out on vertical surfaces. Unfortunately, for VA2, VNA4 and VNA5 the genome sequencing was unsuccessful due to the low concentrations.

#### Fungi

Fungi identification is presented in Table 2. Fungal communities showed the same trend whatever the position with a predominance of Lecanoromycetes class (48.8% for VA1, 38.2% for VA3 and 67.7% for VNA6) followed by Dothiedomycetes class (24.6% for VA1, 14.8% for VA3 and 18.2% for VNA6). At VNA6 position, two other classes were significant: Eurotiomycetes with 3.3% and Sordiariomcetes with 2.2%. Some fungal species were particularly well represented on all positions such as *Teloschistaceae sp*. (VA1: 4.4%, VA3 12.3% and VNA6 50.1%), *Vermiconia calcicola* (VA1 18.7% and VNA6 16.8%), *Solenospora sp*. (VA1 35.2% and VNA6 1.5%). Other species were present in significant proportions according to their position such as *Catillaria lenticularis*, *Pleosporales sp*. for VA1 position, *Lecanorales sp*. and *Cladosporium delicatulum* for VA3 position or *Verrucariaceae sp*. and *Stilbella sp*. for VNA6 position.

**Table 2:** Percent of fungi species identify for each sample. ‘Others’ corresponds to non-identified species.

Sample	Phylum	Class	Species	Relative abundance
VA1	Ascomycota	Dothiedomycetes		**48.8**
			*Pleosporales sp*.	4.4
			*Vermiconia calcicola*	35.2
			*Candelariella aurella*	4.3
			*Catillaria lenticularis*	2.3
			*Others*	2.6
		Lecanoromycetes		**24.6**
			*Solenopsora sp*.	18.7
			*Teloschisteae sp*.	3.2
			*Others*	2.7
VA3	Ascomycota	Lecanoromycetes		**38.2**
			*Lecanorales sp*.	21.8
			*Teloschisteae sp*.	12.8
			*Others*	3.6
		Dothiedomycetes		**14.8**
			*Clasporium delicatulum*	5.9
			*Others*	8.9
VNA6	Ascomycota	Lecanoromycetes		**67.7**
			*Teloschisteae sp*.	50.1
			*Solenopsora sp*.	1.5
			*Others*	16.1
		Dothiedomycetes		**18.2**
			*Vermiconoia calcicola*	16.8
			*Others*	1.4
		Eurotiomycetes		**3.3**
			*Verrucariaceae sp*	2.7
			*Others*	0.6
		Sordiariomycetes		**2.2**
			*Stilbella sp*	2.2

#### Bacteria

For bacteria (Table 3), as a function of their positions, more classes were represented on VA1 and VNA6 position with predominance of Alphaproteobacteria (54% and 61.8%, respectively). In these positions, five other classes were represented: Deinococci, Actinobacteria, Cyanobacteria, Abditibacteria and Bacteroidia. By contrast, for VA3, the major class present is Gammaproteobacteria with 87.5% followed with Alphaproteobacteria with 8.1%. In VA1 and VNA6 positions, genera Sphingomonas and Truepera are well represented with 28.2% and 24.9%, respectively, for VA1 and 28.4% and 9.3% for VNA6 positions. Other genera are present with proportions below 10% such as Rubellimicrobium, MN 122.2a, Acidiphilium, Abditibacterium, Aureimonas and Friedmanniella. VA3 has three major genera Pseudomonas (83.4%), Janthinobacterium (2%) and Sphingomonas (2.5%). The Cyanobacteria identification at genera level was not possible on these samples.

**Table 3:** Percent of bacteria genus identify for each sample. ‘Others’ corresponds to non-identified species.

Sample	Phylum	Class	Genus	Relative abundance
VA1	Proteobacteria	Alphaproteobacteria		**54**
			*Sphingomonas*	28.2
			*Rubellimicrobium*	6.5
			*MN 122*.*2a*	5.2
			*Acidiphilium*	3.6
			*Others*	10.5
		Actinobacteria		7.5
	Deinococcota	Deinococci		**24.9**
			*Truepera*	24.9
	Cyanobacteria	Cyanobacteria		**7.3**
	Abditibacteriota	Abditibacteria		**4.8**
			*Abditibacterium*	4.8
VA3	Proteobacteria	Gammaproteobacteria		**87.5**
			*Pseudomonas*	83.4
			*Janthinobacterium*	2
			*Others*	2.1
		Alphaproteobacteria		**8.1**
			*Sphingomonas*	2.5
			*Others*	5.6
VNA6	Proteobacteria	Alphaproteobacteria		**61.8**
			*Sphingomonas*	28.4
			*Rubellimicrobium*	7
			*MN 122*.*2a*	3.2
			*Acidiphillum*	3.9
			*Aureimons*	7.5
			*Others*	11.8
	Actinobacteriota	Actinobacteria		**11.6**
			*Fiedmaniella*	2
			*Actinoplanes*	2.2
			*Others*	7.4
	Deinococcota	Deinococci		**9.3**
			*Truepera*	9.3
	Cyanobacteria	Cyanobacteria		**5.7**
	Abditibacteriota	Abditibacteria		**3.9**
			*Abditibacterium*	3.9
	Bacteroidota	Bacteroida		**4.8**

#### Alpha-diversity

Different indices relative to the Alpha-diversity have been calculated (Table 4). The three positions: VA1, VA3 and VNA6 showed similar Shannon’s and Simpson’s diversity index for fungi (3.05, 3.04 and 3.02, respectively, for Shannon’s index and 0.87, 0.87 and 0.88 for Simpson’s index). These values show that the microbial communities are relatively similar for sheltered and non-sheltered samples. Unlike fungi, bacteria diversity differed according to their position. VA1 and VNA6 showed higher Shannon’s and Simpson’s index than VA3 (3.51, 4.39 and 2.24, respectively, for Shannon’s index and 0.93, 0.97 and 0.71 for Simpson’s index). Therefore, there is an influence of the sheltered or not situation on the bacteria diversity. To summarize, the fungal species diversity for these positions are equivalent and the bacterial species diversity is higher for VA1 and VNA6 positions compared to VA3.

**Table 4:** Microbial diversity index for VA1, VA3 and VNA6 positions.

**Fungi**							
Beta-diversity				Alpha-diversity			
	VA1	VA3	VNA6	**Observed**	**Chao1**	**Shannon**	**Simpson**
VA1	0	0.95	0.77	311	386.04	3.05	0.87
VA3		0	0.87	439	489.9	3.04	0.87
VNA6			0	313	366.47	3.02	0.88
**Bacteria**							
Beta-diversity				Alpha-diversity			
	VA1	VA3	VNA6	**Observed**	**Chao1**	**Shannon**	**Simpson**
VA1	0	0.94	0.54	260	300.83	3.51	0.93
VA3		0	0.89	312	369	2.24	0.71
VNA6			0	355	370	4.39	0.97

#### Beta-diversity

Fungal Bray Curtis dissimilarity (Table 4) had high values, about 0.77 for VA1-VNA6 and 0.95 for VA1 and VA3, which indicated a different fungal composition on each position. For bacteria, Bray-Curtis dissimilarity was 0.54 for VA1-VNA6 and 0.94 for VA1-VA3. This shows that bacterial community were very different between VA1 and VA3, unlike VA1 and VNA6 that had intermediate similarity.

### Correlation between biological measurements and environmental parameters

Pearson correlation matrices (Table 5) were calculated between different independent meteorological parameters (daily mean temperature, daily mean relative humidity, daily cumulative rainfall) and green algae and cyanobacteria daily concentrations for vertical positions to determine which factors control the presence of algae and cyanobacteria. Results show that there is a good correlation between algae and cyanobacteria concentrations, which highlights that they have a similar evolution and relatively similar values. Concerning meteorological parameters, there is a good correlation of algae and cyanobacteria concentration with RH, especially for VA3, VNA6 that are the locations that remain the wettest. The correlation with rainfall is positive but less significant. On the contrary, the correlation with temperature is negative.

**Table 5:** Pearson correlation matrix realized on 6 samples of different meteorological parameters and benthic microorganisms concentration in vertical positions.

VA1-VA2						VNA4-VNA5					
	T°	RH	P	Algae	Cyano		T°	RH	P	Algae	Cyano
T°	1	-0.71	-0.02	-0.34	-0.42	T°	1	-0.71	-0.02	-0.32	-0.42
RH		1	0.29	0.57	0.55	RH		1	0.29	0.48	0.46
P			1	0.29	0.14	P			1	0.39	0.32
Algae				1	0.64	Algae				1	0.88
Cyano					1	Cyano					1
VA3-VNA6											
	T°	RH	P	Algae	Cyano						
T°	1	-0.71	-0.02	-0.54	-0.64						
RH		1	0.29	0.73	0.79						
P			1	0.19	0.22						
Algae				1	0.95						
Cyano					1						

T°: daily mean temperature in Celsius degree, HR: Daily mean relative humidity in percentage, P: daily cumulative rainfall in millimeter, Algae: green algae daily concentration in μg/cm^2^ and Cyano: cyanobacteria daily concentration in μg/cm^2^.

Based on this correlation matrix, a dose-response function (DRF) that correlates meteorological parameters and the biological colonization on vertical surfaces was developed. On horizontal surfaces, contamination can occur. It was also decided to focus on green algae concentration as cyanobacteria concentration is relatively similar. The dataset is given in S1 Table. Then, the green algae concentration as a function of RH (Fig 7) was fitted using a power law and only considering the data for days without rain:

$$\left[green\;algae\right]=a*{RH}^{b}$$ (1)

Four sets of data were used separately: VA1 and VA2; VA3; VNA4 and VNA5; VNA6. Parameters of Eq (1) are given in Table 6.

**Table 6:** Fitting parameters of green algae concentration as a function of relative humidity (RH) for not-rainy days using a power law (Eq (1)): A, a-range for a 95% confidence interval, b, b-range for a 95% confidence interval, R^2^.

Position	a	a-range (95%)	b	b-range (95%)	R^2^
VA1-VA2	0.507	(0.376;0.639)	7.83	(5.73;9.94)	0.7056
VNA4-VNA5	0.096	(0.052;0.140)	10.00	(5.66;14.34)	0.4444
VA3	1.494	(1.017;1.971)	5.40	(3.33;7.48)	0.7111
VNA6	11.78	(9.52;14.04)	12.41	(10.38;14.44)	0.9364

As green algae concentration is generally higher for rainy days, the rain parameter was added in the DRF:

$$\left[green\;algae\right]=a*{RH}^{b}+\frac{rain}{c}$$ (2)

Different values of c were tested to fit the data. It was noted that c is around 20 in fall-winter and 40 for spring. This is consistent with previous observations that rain leads to higher biological development in winter. Thus, as there is approximately a factor 2 between average temperature (T) during the spring period (16.4°C) and during the fall-winter (7.9°C), c was estimated to be equal to 2.5 / T to take into account this seasonal effect:

$$\left[green\;algae\right]=a*{RH}^{b}+\frac{rain}{2.5*T}$$ (3)

Therefore, 4 dose-response functions were established for the 4 situations: sheltered from tree but exposed to sun (VA1-VA2), sheltered from tree and few exposed to sun (VA3), not sheltered and exposed to sun (VNA4-VNA5), not sheltered and close to soil (VNA6), respectively:

$$\left[green\;algae\right]=0.5*{RH}^{7.8}+\frac{rain}{2.5*T}$$ (4)

$$\left[green\;algae\right]=1.5*{RH}^{5.4}+\frac{rain}{2.5*T}$$ (5)

$$\left[green\;algae\right]=0.1*{RH}^{10.0}+\frac{rain}{2.5*T}$$ (6)

$$\left[green\;algae\right]=11.8*{RH}^{12.4}+\frac{rain}{2.5*T}$$ (7)

**Fig 6:**
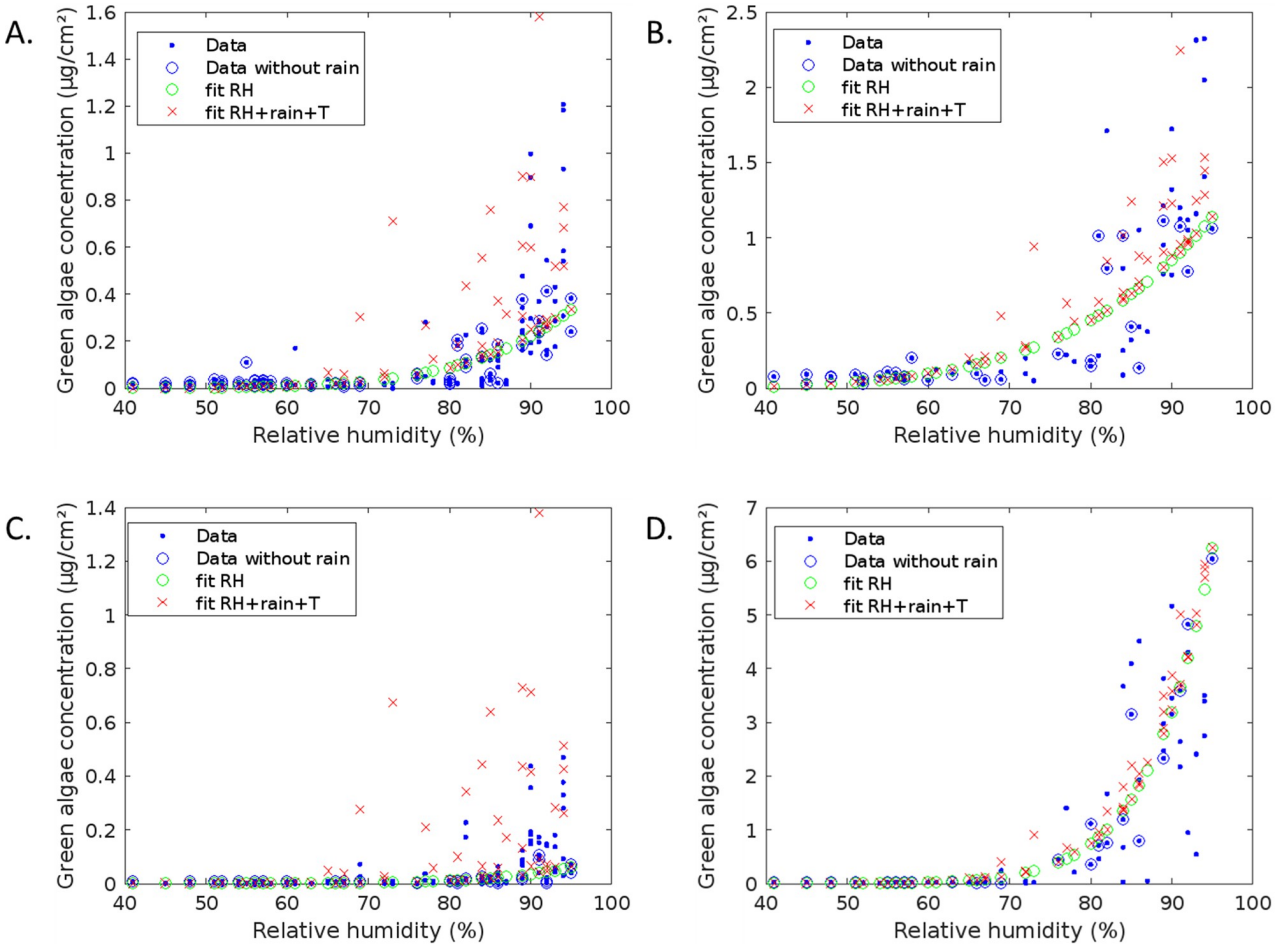
Green algae concentration as a function of RH. Measured data (blue points = all, blue circles = only data for day without rain) and data calculated using DRF by considering only RH (Eq (1), Table 6, green circles) or RH, rain and T (Eq (3), red crosses) for each situation: A. VA1 and VA2 (Eq (3) becomes Eq (4)); B. VA3 (Eq (5); C. VNA4 and VNA5 (Eq (6); D. VNA6 (Eq (7)).

Fig 6 shows that the results are the best for VNA6 and VA3. This can be explained by a higher and longer humidity of these zones (close to the ground), which leads to lower contrast between wet and dry periods. This can be seen by the lower gap between data with and without rain. However, the fitting of the data is quite good.

**Fig 7:**
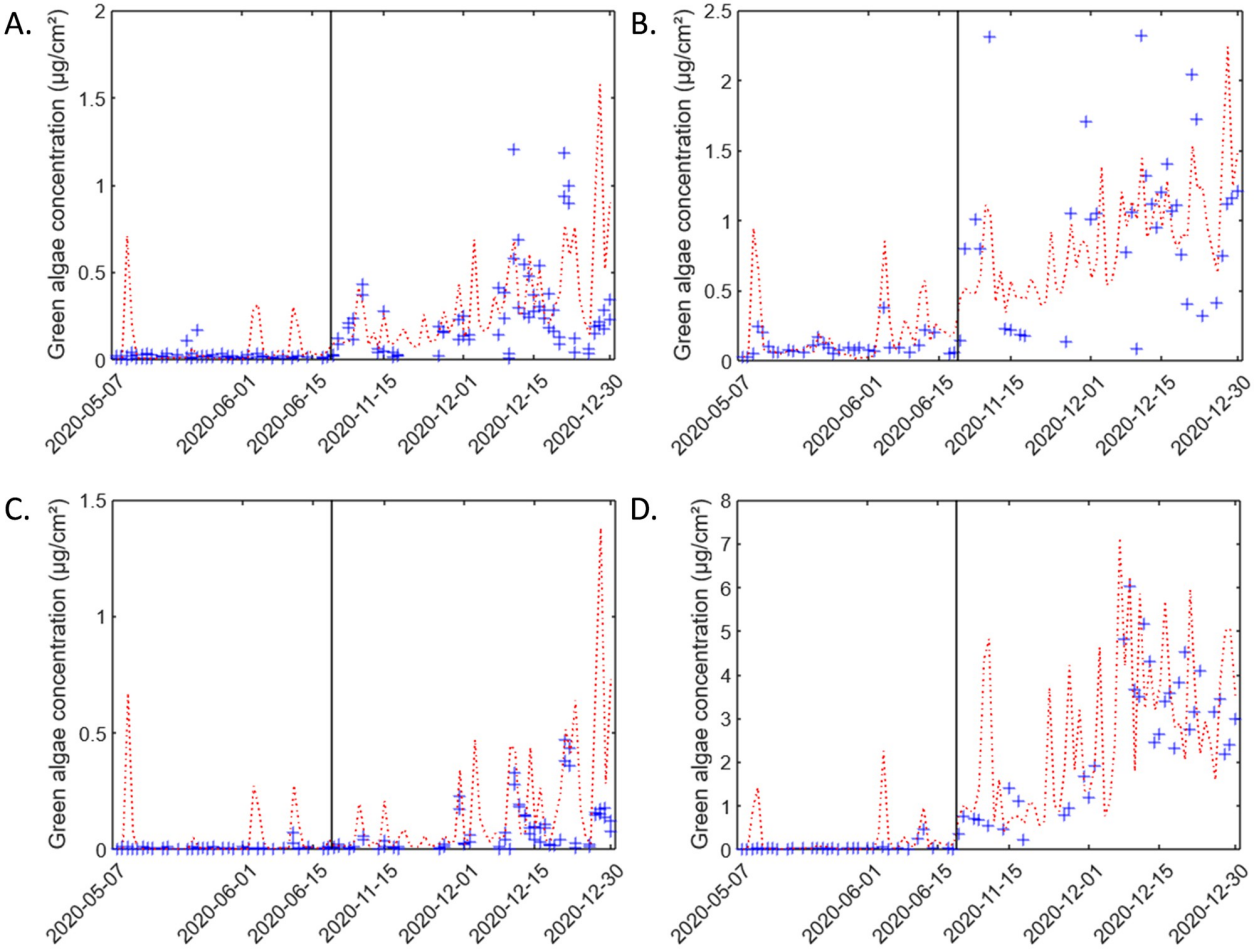
Temporal evolution of green algae concentration. Measured data (blue points) and data calculated using DRF (red line) for each situation: A. VA1 and VA2 (Eq (4)); B. VA3 (Eq (5); C. VNA4 and VNA5 (Eq (6); D. VNA6 (Eq (7)).

Then, the calculated values have been compared to measured data as a function of time (Fig 7). Results are relatively consistent. For spring, some peaks of green algae can be explained by meteorological parameters, but some cannot. Generally, in spring some increases of the green algae concentration are predicted by the DRF and not measured by BenthoTorch. In some cases, this is because the measurement has been done before the rain event. For the fall-winter period, the general trend and the variations are well simulated by the DRF, sometimes with a slight shift.

## Discussion

The objective of this study was to improve the understanding about the climate influence on microbial development and their dynamic over time in order to understand the impact of climate (change) on plaster and tiles deteriorations under temperate climate during two contrasted seasons: spring and fall-winter.

### Influence of the climate on algae and cyanobacteria concentrations

The cumulative concentration in microorganisms (Fig 5) vary according to the season, to their location on the wall, to the orientation (vertical vs. horizontal) and to the sheltered or unsheltered from the tree situation. This can be explained by differences of time of wetness that is dependent on temperature, rain amount, relative humidity and insolation. The differences of behavior as a function of microorganisms gives also some clues.

Globally, for vertical samples, the microorganisms concentrations are systematically higher in winter than in spring. Regarding the environmental data registered for both periods (see §3.2.), the temperature is lower in winter than in spring, whereas precipitation (133.4 mm after 55 days in fall-winter vs. 57.8 mm after 42 days in spring) and RH (78% of time where RH > 80% in fall-winter vs. 19% of time in spring) are higher. This indicates that humidity favors the microbial development. These results are consistent with the literature [19].

For horizontal samples, after each rainfall event, a proliferation of microorganisms was observed whatever the season. However, it can result from the contamination of the paving stones close to the measured one. The comparison with a previously bleach-cleaned tile confirmed that this proliferation is caused by the remobilization of microorganisms present on the stones.

For vertical samples, the results show also differences according to the sheltered from the tree or not situation. VA samples have generally higher values that VNA samples except for VNA6 that is close to the ground and can be affected by rising damp. This shows that the tree contributes to keep the surface humid. Beyond temperature, rain and humidity that are key parameters for the proliferation of microorganisms, local factors such as insolation must also be taken into account [20]. As it is a parameter difficult to quantify on cumulative time periods, we can qualitatively distinguish two microclimates. The first one the north façade under the tree (VA measurements) is associated to a “shaded microclimate” and is characterized by an important development of microorganisms. Locally, the temperature is lower and the wall surface remains wetter longer. The second microclimate corresponds to a “sunny microclimate” (VNA measurements) with the façade exposed at south and a lower development of microorganisms. A “sunny microclimate” can induce more thermal stress on the surface leading to a decrease in the concentrations of cyanobacteria and green algae because of direct solar exposition inducing higher rapid temperature variations, especially during sunny days [21]. Ortega-Morales et al. 2013 [22] have shown that solar irradiation could be the most important factor to microbial development due to a high radiation reducing the time of wetness and increasing the UV exposure.

Cyanobacteria concentrations are less sensitive to the season than green algae. Also, in spring, cyanobacteria concentrations were higher than green algae concentrations whatever the positions (horizontal and vertical), whereas in winter, green algae concentrations were higher than those in cyanobacteria excepted for some vertical positions. The difference between cyanobacteria and green algae can be explained by the ability of cyanobacteria to survive and recover faster from desiccation than algae [19]. Their predominance in spring can also be explained by their ability to resist to high temperature variations [8]. They are less sensible to dryness and to intense solar radiation [20]. These results are in accordance with those of Quagliarini et al. 2019 [23] that have highlighted a good correlation between green algae concentration in low temperature and cyanobacteria concentration in higher temperature.

### Expected effects of the climate change on the microorganism development dynamics

Based on the examination of data and on statistical analyses, dose-response functions have been established (Eqs (4) to (7)) for green algae concentration. They take into account global parameters: RH, rain and temperature. However, the results show significant differences as a function of the microclimate (insolation that depends on potential shelter such as the tree, proximity to the ground that can be affected by rising damp, etc.). At this stage, these local effects that are difficult to quantify using general and averaged parameters are integrated using specific fitted parameters for each situation or microclimate.

These DRF have of course to be completed by new measurements campaigns and are at this stage relatively specific. However, they confirmed the influence of key parameters, they give a first estimation of the contribution of each of them and they could be used to assess the potential effects of climate change. For example, the Parisian climate during 1901–2000 period showed an increase of annual mean temperature of 1.6°C and precipitation of 13% with an acceleration on second part of the century (Parisian climate agency 2021). Short-duration extreme precipitations will be intensified for all scenarios [24]. The Volume 4 of the report "France’s Climate in the 21st Century" [25] presents climate change scenarios in France up to 2100. By 2021–2050, the models predict an increase in average temperature between 0.6 and 1.3°C compared to the reference average calculated over the period 1976–2005 and a slight increase in average precipitation both in summer and winter (between 0 and 0.42 mm/day on average over France). Relative humidity previsions show that in winter, relative humidity will increase in December–February period [26]. As temperature and rain have contradictory effects, it is difficult to precisely anticipate the algal development. However, it is probable that the increase of rain and humidity will lead to an increase of the cumulative microorganisms concentration. This biological proliferation will add to other harmful effects, such as salt crystallization [27].

### The biodiversity on plaster and composite materials

In this paper, we focused on the monitoring of green algae and cyanobacteria concentration. However, literature data have shown the influence of a huge kind of microorganisms for the deterioration of buildings. Our analysis of the bacterial and fungal diversities using next generation technique permits to support the literature data. At our knowledge, no studies have reported NGS analysis on plaster of modern building and on the influence of microclimate on microbial diversity. The calculation of the Beta-diversity for each position have highlighted an effect on the microclimate on the microbial diversity whatever the kind of microorganisms (fungi and bacteria). VA1 and VA3 positions have very similar microbial community with Beta-diversity index of 0.95 for fungi and 0.94 for bacteria. The comparison of these positions with VNA6 shows lower dissimilarity index, which highlights the microbial communities difference between VA1, VA3 and VNA6. For fungi, two species are added on VNA6 in comparison with VA1 and VA3: *Verrucariaceae sp*. and *Stibella sp*. The first one is well known to be a lichenized-fungi, in symbiont often with green algae, and lives frequently on rock surface [28]. *Stibella sp*. belongs to Sordariomycetes that are essentially parasites and are involved in decomposition and nutrient cycling [29].

Moreover, bacteria present on building surface are common to microbial community observed on limestone around the world [30, 31]. Genera Truepera and Rubellimicrobium have been observed and are known to be alkanophilic and largely present on limestone surface [32]. Sphingomonas genus, dominant on vertical surfaces, has been identified on different rock type surfaces [15, 33]. Abditibacterium genus has been identified on stone surfaces of Chinese monuments [15] and on different cemetery locations [32]. Gammaproteobacteria is the most abundant phylum on VA3, representing 87.5%. Among this phylum, Pseudomonas genus is the most represented, followed by Sphingomonas and Janthinobacterium genera. Pseudomonas are ubiquist. Janthinobacterium can produce blue-violet pigment, visible on the surface. Thus, the bacterial and fungal communities compositions analyses on plaster is very similar to what is observed on limestone that is a very common material of more ancient buildings.

## Conclusion

This study is a first attempt to monitor the biological development on building surfaces at the daily scale as a function of meteorological parameters to assess the reactivity and the dynamics of this development. The results show that BenthoTorch is a suitable tool to perform this fine monitoring as peaks of green algae and cyanobacteria occur a few hours after a rain event. The data also highlights that the colonization by these photolithotrophic microorganisms is more important in winter than in spring and is very dependent of water availability (relative humidity, rain and capillarity rise). From the data, it was possible to implement first dose-response functions that correlate the microbial development to the environmental parameters. They need to be improved to account for the microclimate effect.

## Supporting information

S1 Table Meteorological data and microbial concentration for each positions. (temperature T in °C, relative humidity RH in %, rain quantity in mm) and concentrations of green algae (‘algae’) and cyanobacteria (‘cyano’) for each day of the 2 measurements campaigns.
(DOCX)
