# Supplementary material for: Influence of climatic factors on cyanobacteria and green algae development on building surface
Source: PLoS One. 2023 Mar 6;18(3):e0282140. doi: 10.1371/journal.pone.0282140 (PMC9987821; doi:10.1371/journal.pone.0282140)
Supplement: S1 Table — (temperature T in °C, relative humidity RH in %, rain quantity in mm) and concentrations of green algae (‘algae’) and cyanobacteria (‘cyano’) for each day of the 2 measurements campaigns. (DOCX) [file pone.0282140.s001.docx]

SUPPLEMENTARY MATERIALS

Table S1. Meteorological data (temperature T in °C, relative humidity RH in %, rain quantity in mm) and concentrations of green algae (‘algae’) and cyanobacteria (‘cyano’) for each day of the 2 measurements campaigns.

| Date | T | RH | Rain | VA1 |  | VA2 |  | VA3 |  | VNA4 |  | VNA5 |  | VNA6 |  | HA1 |  | HNA2 |  | HBS |  | HBP |  |
| --- | --- | --- | --- | --- | --- | --- | --- | --- | --- | --- | --- | --- | --- | --- | --- | --- | --- | --- | --- | --- | --- | --- | --- |
|  | °C | % | mm | cyano | algae | cyano | algae | cyano | algae | cyano | algae | cyano | algae | cyano | algae | cyano | algae | cyano | algae | cyano | algae | cyano | algae |
| 05/07/20 | 15.6 | 45 | 0.0 | 0.020 | 0.020 | 0.010 | 0.010 | 0.060 | 0.030 | 0.020 | 0.000 | 0.020 | 0.000 | 0.060 | 0.020 | 0.065 | 0.020 | 0.055 | 0.010 | nd | nd | nd | nd |
| 05/08/20 | 17.5 | 52 | 0.0 | 0.020 | 0.020 | 0.010 | 0.010 | 0.020 | 0.030 | 0.020 | 0.010 | 0.020 | 0.000 | 0.040 | 0.010 | 0.050 | 0.005 | 0.055 | 0.010 | nd | nd | nd | nd |
| 05/09/20 | 18.3 | 73 | 30.5 | 0.070 | 0.020 | 0.010 | 0.000 | 0.110 | 0.050 | 0.020 | 0.000 | 0.020 | 0.010 | 0.050 | 0.020 | 0.060 | 0.020 | 0.055 | 0.010 | nd | nd | nd | nd |
| 05/10/20 | 16.5 | 84 | 2.0 | 0.080 | 0.040 | 0.010 | 0.020 | 0.280 | 0.250 | 0.010 | 0.000 | 0.020 | 0.000 | 0.050 | 0.030 | 0.075 | 0.030 | 0.035 | 0.015 | nd | nd | nd | nd |
| 05/11/20 | 9.2 | 58 | 0.0 | 0.220 | 0.030 | 0.020 | 0.010 | 0.220 | 0.200 | 0.020 | 0.000 | 0.020 | 0.000 | 0.060 | 0.020 | 0.065 | 0.020 | 0.050 | 0.025 | nd | nd | nd | nd |
| 05/12/20 | 9.0 | 56 | 0.0 | 0.130 | 0.030 | 0.010 | 0.010 | 0.110 | 0.100 | 0.020 | 0.000 | 0.020 | 0.010 | 0.050 | 0.030 | 0.060 | 0.020 | 0.040 | 0.015 | nd | nd | nd | nd |
| 05/13/20 | 9.9 | 56 | 0.0 | 0.127 | 0.033 | 0.010 | 0.007 | 0.087 | 0.063 | 0.020 | 0.007 | 0.017 | 0.007 | 0.043 | 0.023 | 0.073 | 0.023 | 0.043 | 0.017 | nd | nd | nd | nd |
| 05/14/20 | 9.9 | 52 | 0.0 | 0.160 | 0.030 | 0.010 | 0.010 | 0.060 | 0.063 | 0.013 | 0.007 | 0.017 | 0.003 | 0.050 | 0.017 | 0.050 | 0.020 | 0.040 | 0.017 | nd | nd | nd | nd |
| 05/15/20 | 11.3 | 53 | 0.0 | nd | nd | nd | nd | nd | nd | nd | nd | nd | nd | nd | nd | nd | nd | nd | nd | nd | nd | nd | nd |
| 05/16/20 | 13.0 | 57 | 0.0 | 0.143 | 0.020 | 0.010 | 0.013 | 0.093 | 0.080 | 0.017 | 0.007 | 0.017 | 0.003 | 0.043 | 0.013 | 0.063 | 0.027 | 0.040 | 0.013 | nd | nd | nd | nd |
| 05/17/20 | 14.5 | 57 | 0.0 | 0.087 | 0.033 | 0.010 | 0.013 | 0.090 | 0.070 | 0.017 | 0.003 | 0.017 | 0.010 | 0.037 | 0.023 | 0.057 | 0.023 | 0.050 | 0.020 | nd | nd | nd | nd |
| 05/18/20 | 16.3 | 58 | 0.0 | nd | nd | nd | nd | nd | nd | nd | nd | nd | nd | nd | nd | nd | nd | nd | nd | nd | nd | nd | nd |
| 05/19/20 | 16.9 | 57 | 0.0 | 0.097 | 0.030 | 0.013 | 0.017 | 0.073 | 0.060 | 0.020 | 0.000 | 0.020 | 0.003 | 0.033 | 0.027 | 0.060 | 0.030 | 0.047 | 0.023 | nd | nd | nd | nd |
| 05/20/20 | 19.9 | 62 | 0.0 | nd | nd | nd | nd | nd | nd | nd | nd | nd | nd | nd | nd | nd | nd | nd | nd | nd | nd | nd | nd |
| 05/21/20 | 23.1 | 55 | 0.0 | 0.073 | 0.020 | 0.010 | 0.013 | 0.090 | 0.110 | 0.013 | 0.000 | 0.017 | 0.003 | 0.040 | 0.023 | 0.057 | 0.023 | 0.053 | 0.023 | nd | nd | nd | nd |
| 05/22/20 | 20.3 | 65 | 2.4 | 0.117 | 0.037 | 0.010 | 0.007 | 0.187 | 0.173 | 0.017 | 0.003 | 0.020 | 0.010 | 0.047 | 0.023 | 0.070 | 0.033 | 0.373 | 0.393 | nd | nd | nd | nd |
| 05/23/20 | 15.3 | 61 | 0.2 | 0.157 | 0.007 | 0.020 | 0.013 | 0.197 | 0.123 | 0.017 | 0.000 | 0.013 | 0.007 | 0.053 | 0.030 | 0.060 | 0.020 | 0.057 | 0.017 | nd | nd | nd | nd |
| 05/24/20 | 15.0 | 66 | 0.0 | 0.077 | 0.017 | 0.013 | 0.020 | 0.147 | 0.097 | 0.017 | 0.007 | 0.013 | 0.007 | 0.053 | 0.017 | 0.057 | 0.017 | 0.050 | 0.017 | nd | nd | nd | nd |
| 05/25/20 | 17.4 | 60 | 0.0 | 0.097 | 0.020 | 0.013 | 0.013 | 0.083 | 0.053 | 0.017 | 0.000 | 0.047 | 0.007 | 0.033 | 0.023 | 0.053 | 0.013 | 0.040 | 0.013 | nd | nd | nd | nd |
| 05/26/20 | 19.1 | 54 | 0.0 | 0.070 | 0.027 | 0.013 | 0.017 | 0.120 | 0.080 | 0.013 | 0.010 | 0.013 | 0.000 | 0.030 | 0.013 | 0.050 | 0.010 | 0.040 | 0.013 | nd | nd | nd | nd |
| 05/27/20 | 19.5 | 52 | 0.0 | nd | nd | nd | nd | nd | nd | nd | nd | nd | nd | nd | nd | nd | nd | nd | nd | nd | nd | nd | nd |
| 05/28/20 | 17.8 | 51 | 0.0 | 0.073 | 0.037 | 0.020 | 0.017 | 0.117 | 0.093 | 0.013 | 0.007 | 0.020 | 0.003 | 0.030 | 0.033 | 0.050 | 0.013 | 0.043 | 0.010 | nd | nd | nd | nd |
| 05/29/20 | 18.4 | 41 | 0.0 | 0.087 | 0.020 | 0.017 | 0.017 | 0.097 | 0.077 | 0.013 | 0.007 | 0.013 | 0.007 | 0.033 | 0.027 | 0.057 | 0.013 | 0.043 | 0.013 | nd | nd | nd | nd |
| 05/30/20 | 19.1 | 45 | 0.0 | 0.093 | 0.020 | 0.013 | 0.007 | 0.093 | 0.093 | 0.017 | 0.000 | 0.020 | 0.003 | 0.033 | 0.027 | 0.057 | 0.013 | 0.047 | 0.007 | nd | nd | nd | nd |
| 05/31/20 | 18.5 | 43 | 0.0 | nd | nd | nd | nd | nd | nd | nd | nd | nd | nd | nd | nd | nd | nd | nd | nd | nd | nd | nd | nd |
| 06/01/20 | 20.2 | 48 | 0.0 | 0.070 | 0.013 | 0.010 | 0.010 | 0.087 | 0.077 | 0.013 | 0.010 | 0.023 | 0.003 | 0.037 | 0.033 | 0.050 | 0.013 | 0.050 | 0.013 | nd | nd | nd | nd |
| 06/02/20 | 22.4 | 48 | 0.0 | 0.070 | 0.027 | 0.010 | 0.010 | 0.090 | 0.073 | 0.017 | 0.007 | 0.017 | 0.003 | 0.040 | 0.023 | 0.060 | 0.017 | 0.090 | 0.033 | 0.037 | 0.000 | 0.017 | 0.000 |
| 06/03/20 | 20.1 | 57 | 13.5 | nd | nd | nd | nd | nd | nd | nd | nd | nd | nd | nd | nd | nd | nd | nd | nd | nd | nd | nd | nd |
| 06/04/20 | 12.9 | 87 | 4.7 | 0.310 | 0.017 | 0.033 | 0.033 | 0.383 | 0.377 | 0.023 | 0.007 | 0.023 | 0.003 | 0.083 | 0.050 | 0.120 | 0.083 | 0.067 | 0.043 | 0.023 | 0.000 | 0.030 | 0.000 |
| 06/05/20 | 13.4 | 72 | 0.8 | 0.070 | 0.020 | 0.027 | 0.013 | 0.140 | 0.097 | 0.017 | 0.007 | 0.020 | 0.000 | 0.037 | 0.010 | 0.230 | 0.180 | 0.140 | 0.140 | 0.040 | 0.003 | 0.013 | 0.000 |
| 06/06/20 | 14.1 | 57 | 0.0 | nd | nd | nd | nd | nd | nd | nd | nd | nd | nd | nd | nd | nd | nd | nd | nd | nd | nd | nd | nd |
| 06/07/20 | 14.5 | 63 | 0.0 | 0.077 | 0.017 | 0.017 | 0.010 | 0.153 | 0.093 | 0.020 | 0.003 | 0.020 | 0.000 | 0.040 | 0.023 | 0.053 | 0.010 | 0.140 | 0.143 | 0.043 | 0.000 | 0.017 | 0.000 |
| 06/08/20 | 13.6 | 74 | 0.0 | nd | nd | nd | nd | nd | nd | nd | nd | nd | nd | nd | nd | nd | nd | nd | nd | nd | nd | nd | nd |
| 06/09/20 | 13.7 | 69 | 0.0 | 0.043 | 0.020 | 0.010 | 0.010 | 0.067 | 0.057 | 0.020 | 0.007 | 0.020 | 0.007 | 0.020 | 0.007 | 0.050 | 0.007 | 0.043 | 0.007 | 0.020 | 0.000 | 0.020 | 0.000 |
| 06/10/20 | 15.2 | 62 | 0.0 | nd | nd | nd | nd | nd | nd | nd | nd | nd | nd | nd | nd | nd | nd | nd | nd | nd | nd | nd | nd |
| 06/11/20 | 17.0 | 69 | 11.6 | 0.097 | 0.017 | 0.030 | 0.007 | 0.213 | 0.110 | 0.093 | 0.073 | 0.080 | 0.027 | 0.243 | 0.243 | 0.860 | 0.777 | 0.733 | 0.557 | 0.113 | 0.080 | 0.033 | 0.000 |
| 06/12/20 | 16.2 | 81 | 3.6 | 0.080 | 0.020 | 0.035 | 0.020 | 0.223 | 0.217 | 0.023 | 0.007 | 0.023 | 0.003 | 0.330 | 0.463 | 0.293 | 0.313 | 0.053 | 0.013 | 0.040 | 0.013 | 0.013 | 0.000 |
| 06/13/20 | 18.5 | 64 | 0.0 | nd | nd | nd | nd | nd | nd | nd | nd | nd | nd | nd | nd | nd | nd | nd | nd | nd | nd | nd | nd |
| 06/14/20 | 17.6 | 72 | 0.4 | 0.097 | 0.017 | 0.020 | 0.013 | 0.253 | 0.200 | 0.023 | 0.007 | 0.017 | 0.010 | 0.080 | 0.043 | 0.177 | 0.163 | 0.273 | 0.387 | 0.057 | 0.033 | 0.037 | 0.000 |
| 06/15/20 | 18.1 | 70 | 0.0 | nd | nd | nd | nd | nd | nd | nd | nd | nd | nd | nd | nd | nd | nd | nd | nd | nd | nd | nd | nd |
| 06/16/20 | 18.2 | 69 | 0.0 | nd | nd | nd | nd | nd | nd | nd | nd | nd | nd | nd | nd | nd | nd | nd | nd | nd | nd | nd | nd |
| 06/17/20 | 17.7 | 67 | 0.0 | 0.073 | 0.007 | 0.013 | 0.013 | 0.070 | 0.053 | 0.017 | 0.003 | 0.017 | 0.007 | 0.040 | 0.027 | 0.043 | 0.013 | 0.033 | 0.013 | 0.013 | 0.000 | 0.017 | 0.003 |
| 06/18/20 | 17.6 | 67 | 1.6 | 0.060 | 0.017 | 0.020 | 0.013 | 0.083 | 0.057 | 0.017 | 0.003 | 0.010 | 0.010 | 0.027 | 0.003 | 0.047 | 0.017 | 0.033 | 0.013 | 0.023 | 0.010 | 0.020 | 0.000 |
| 11/05/20 | 6.3 | 80 | 0.0 | 0.193 | 0.030 | 0.060 | 0.023 | 0.217 | 0.147 | 0.020 | 0.007 | 0.020 | 0.010 | 0.260 | 0.353 | 0.200 | 0.097 | 0.100 | 0.080 | 0.037 | 0.023 | nd | nd |
| 11/06/20 | 7.0 | 82 | 0.0 | 0.367 | 0.123 | 0.090 | 0.090 | 0.377 | 0.797 | 0.030 | 0.020 | 0.020 | 0.013 | 0.370 | 0.753 | 0.160 | 0.077 | 0.090 | 0.070 | 0.037 | 0.033 | nd | nd |
| 11/07/20 | 12.2 | 81 | 0.0 | nd | nd | nd | nd | nd | nd | nd | nd | nd | nd | nd | nd | nd | nd | nd | nd | nd | nd | nd | nd |
| 11/08/20 | 13.9 | 81 | 0.0 | 0.390 | 0.207 | 0.087 | 0.180 | 0.453 | 1.013 | 0.030 | 0.003 | 0.020 | 0.007 | 0.377 | 0.713 | 0.307 | 0.400 | 0.177 | 0.363 | 0.060 | 0.107 | nd | nd |
| 11/09/20 | 13.8 | 84 | 0.4 | 0.580 | 0.237 | 0.107 | 0.117 | 0.353 | 0.797 | 0.020 | 0.013 | 0.020 | 0.010 | 0.420 | 0.673 | 0.367 | 0.793 | 0.580 | 0.703 | 0.080 | 0.287 | nd | nd |
| 11/10/20 | 12.6 | 92 | 4.8 | nd | nd | nd | nd | nd | nd | nd | nd | nd | nd | nd | nd | nd | nd | nd | nd | nd | nd | nd | nd |
| 11/11/20 | 11.1 | 93 | 0.4 | 0.547 | 0.430 | 0.127 | 0.370 | 0.777 | 2.313 | 0.040 | 0.043 | 0.057 | 0.057 | 0.257 | 0.543 | 0.613 | 1.053 | 0.193 | 0.463 | 0.087 | 0.360 | nd | nd |
| 11/12/20 | 10.9 | 79 | 0.0 | nd | nd | nd | nd | nd | nd | nd | nd | nd | nd | nd | nd | nd | nd | nd | nd | nd | nd | nd | nd |
| 11/13/20 | 12.5 | 85 | 0.4 | nd | nd | nd | nd | nd | nd | nd | nd | nd | nd | nd | nd | nd | nd | nd | nd | nd | nd | nd | nd |
| 11/14/20 | 13.6 | 76 | 0.0 | 0.120 | 0.060 | 0.057 | 0.040 | 0.187 | 0.230 | 0.030 | 0.003 | 0.020 | 0.000 | 0.483 | 0.453 | 0.310 | 0.170 | 0.110 | 0.090 | 0.043 | 0.083 | nd | nd |
| 11/15/20 | 13.0 | 77 | 6.5 | 0.460 | 0.280 | 0.033 | 0.050 | 0.163 | 0.220 | 0.033 | 0.013 | 0.020 | 0.037 | 0.873 | 1.403 | 1.330 | 1.400 | 0.207 | 0.610 | 0.053 | 0.233 | nd | nd |
| 11/16/20 | 11.0 | 80 | 0.2 | nd | nd | nd | nd | nd | nd | nd | nd | nd | nd | nd | nd | nd | nd | nd | nd | nd | nd | nd | nd |
| 11/17/20 | 11.3 | 80 | 0.0 | 0.123 | 0.040 | 0.017 | 0.017 | 0.153 | 0.183 | 0.020 | 0.013 | 0.020 | 0.007 | 0.580 | 1.117 | 0.253 | 0.110 | 0.097 | 0.057 | 0.030 | 0.027 | nd | nd |
| 11/18/20 | 11.6 | 78 | 1.4 | 0.210 | 0.030 | 0.027 | 0.023 | 0.190 | 0.177 | 0.027 | 0.010 | 0.020 | 0.003 | 0.213 | 0.213 | 0.260 | 0.113 | 0.130 | 0.123 | 0.033 | 0.047 | nd | nd |
| 11/19/20 | 9.1 | 82 | 1.6 | nd | nd | nd | nd | nd | nd | nd | nd | nd | nd | nd | nd | nd | nd | nd | nd | nd | nd | nd | nd |
| 11/20/20 | 6.3 | 82 | 0.0 | nd | nd | nd | nd | nd | nd | nd | nd | nd | nd | nd | nd | nd | nd | nd | nd | nd | nd | nd | nd |
| 11/21/20 | 7.2 | 78 | 0.0 | nd | nd | nd | nd | nd | nd | nd | nd | nd | nd | nd | nd | nd | nd | nd | nd | nd | nd | nd | nd |
| 11/22/20 | 8.3 | 79 | 0.0 | nd | nd | nd | nd | nd | nd | nd | nd | nd | nd | nd | nd | nd | nd | nd | nd | nd | nd | nd | nd |
| 11/23/20 | 9.4 | 91 | 0.4 | nd | nd | nd | nd | nd | nd | nd | nd | nd | nd | nd | nd | nd | nd | nd | nd | nd | nd | nd | nd |
| 11/24/20 | 7.0 | 82 | 0.0 | nd | nd | nd | nd | nd | nd | nd | nd | nd | nd | nd | nd | nd | nd | nd | nd | nd | nd | nd | nd |
| 11/25/20 | 7.5 | 81 | 0.0 | nd | nd | nd | nd | nd | nd | nd | nd | nd | nd | nd | nd | nd | nd | nd | nd | nd | nd | nd | nd |
| 11/26/20 | 6.6 | 86 | 0.0 | 0.330 | 0.187 | 0.017 | 0.023 | 0.130 | 0.137 | 0.020 | 0.000 | 0.027 | 0.007 | 0.623 | 0.800 | 0.133 | 0.057 | 0.117 | 0.087 | 0.027 | 0.027 | nd | nd |
| 11/27/20 | 6.1 | 92 | 0.2 | 0.343 | 0.163 | 0.063 | 0.157 | 0.557 | 1.050 | 0.030 | 0.013 | 0.027 | 0.023 | 0.713 | 0.947 | 0.170 | 0.090 | 0.097 | 0.070 | 0.043 | 0.053 | nd | nd |
| 11/28/20 | 5.5 | 86 | 0.0 | nd | nd | nd | nd | nd | nd | nd | nd | nd | nd | nd | nd | nd | nd | nd | nd | nd | nd | nd | nd |
| 11/29/20 | 2.2 | 90 | 0.0 | nd | nd | nd | nd | nd | nd | nd | nd | nd | nd | nd | nd | nd | nd | nd | nd | nd | nd | nd | nd |
| 11/30/20 | 3.2 | 82 | 2.6 | 0.343 | 0.113 | 0.100 | 0.227 | 0.560 | 1.710 | 0.537 | 0.227 | 0.297 | 0.173 | 1.240 | 1.673 | 1.635 | 0.840 | 0.463 | 0.513 | 0.130 | 0.443 | nd | nd |
| 12/01/20 | 6.8 | 84 | 0.0 | 0.340 | 0.133 | 0.140 | 0.253 | 0.510 | 1.010 | 0.037 | 0.027 | 0.043 | 0.020 | 0.387 | 1.190 | 0.577 | 0.897 | 0.580 | 0.763 | 0.077 | 0.337 | nd | nd |
| 12/02/20 | 6.8 | 86 | 0.6 | 0.480 | 0.143 | 0.080 | 0.117 | 0.597 | 1.053 | 0.050 | 0.063 | 0.063 | 0.030 | 0.853 | 1.927 | 0.327 | 0.567 | 0.167 | 0.407 | 0.063 | 0.253 | nd | nd |
| 12/03/20 | 6.3 | 92 | 6.7 | nd | nd | nd | nd | nd | nd | nd | nd | nd | nd | nd | nd | nd | nd | nd | nd | nd | nd | nd | nd |
| 12/04/20 | 5.4 | 79 | 1.6 | nd | nd | nd | nd | nd | nd | nd | nd | nd | nd | nd | nd | nd | nd | nd | nd | nd | nd | nd | nd |
| 12/05/20 | 4.6 | 82 | 0.6 | nd | nd | nd | nd | nd | nd | nd | nd | nd | nd | nd | nd | nd | nd | nd | nd | nd | nd | nd | nd |
| 12/06/20 | 3.2 | 88 | 0.0 | nd | nd | nd | nd | nd | nd | nd | nd | nd | nd | nd | nd | nd | nd | nd | nd | nd | nd | nd | nd |
| 12/07/20 | 2.2 | 96 | 0.0 | nd | nd | nd | nd | nd | nd | nd | nd | nd | nd | nd | nd | nd | nd | nd | nd | nd | nd | nd | nd |
| 12/08/20 | 2.1 | 92 | 0.0 | 0.243 | 0.143 | 0.150 | 0.413 | 0.460 | 0.777 | 0.030 | 0.003 | 0.027 | 0.010 | 2.367 | 4.820 | 0.260 | 0.333 | 0.147 | 0.180 | 0.140 | 0.063 | nd | nd |
| 12/09/20 | 1.6 | 95 | 0.0 | 0.243 | 0.240 | 0.097 | 0.383 | 0.490 | 1.060 | 0.083 | 0.070 | 0.097 | 0.040 | 2.217 | 6.040 | 0.480 | 0.887 | 0.267 | 0.617 | 0.100 | 0.423 | nd | nd |
| 12/10/20 | 4.7 | 84 | 5.0 | 0.133 | 0.033 | 0.017 | 0.010 | 0.117 | 0.087 | 0.023 | 0.003 | 0.020 | 0.003 | 1.507 | 3.673 | 0.137 | 0.057 | 0.033 | 0.033 | 0.077 | 0.053 | nd | nd |
| 12/11/20 | 8.4 | 94 | 7.8 | 0.280 | 0.583 | 0.450 | 1.207 | 0.827 | 2.323 | 0.265 | 0.330 | 0.360 | 0.280 | 2.730 | 3.497 | 1.137 | 1.140 | 0.617 | 0.573 | 0.147 | 0.677 | nd | nd |
| 12/12/20 | 8.1 | 90 | 0.6 | 0.203 | 0.297 | 0.260 | 0.690 | 0.527 | 1.320 | 0.237 | 0.193 | 0.197 | 0.183 | 3.183 | 5.160 | 1.323 | 1.363 | 0.617 | 0.887 | 0.160 | 0.697 | nd | nd |
| 12/13/20 | 5.9 | 92 | 0.4 | 0.210 | 0.263 | 0.237 | 0.543 | 0.483 | 1.117 | 0.180 | 0.140 | 0.153 | 0.147 | 2.203 | 4.307 | 1.260 | 1.340 | 0.587 | 0.843 | 0.140 | 0.543 | nd | nd |
| 12/14/20 | 10.2 | 89 | 10.3 | 0.220 | 0.247 | 0.197 | 0.477 | 0.450 | 0.953 | 0.090 | 0.090 | 0.053 | 0.067 | 1.787 | 2.467 | 0.707 | 0.993 | 0.583 | 0.817 | 0.100 | 0.473 | nd | nd |
| 12/15/20 | 9.4 | 91 | 1.2 | 0.377 | 0.280 | 0.153 | 0.370 | 0.507 | 1.200 | 0.073 | 0.097 | 0.063 | 0.040 | 1.693 | 2.640 | 0.917 | 1.307 | 0.427 | 0.873 | 0.147 | 0.707 | nd | nd |
| 12/16/20 | 7.7 | 94 | 4.0 | 0.433 | 0.540 | 0.087 | 0.307 | 0.567 | 1.407 | 0.067 | 0.093 | 0.067 | 0.080 | 1.353 | 3.393 | 1.143 | 1.600 | 0.280 | 0.977 | 0.153 | 0.917 | nd | nd |
| 12/17/20 | 8.9 | 91 | 0.0 | 0.217 | 0.237 | 0.123 | 0.287 | 0.470 | 1.073 | 0.073 | 0.090 | 0.080 | 0.107 | 1.813 | 3.583 | 1.343 | 1.930 | 0.607 | 1.360 | 0.123 | 0.903 | nd | nd |
| 12/18/20 | 7.9 | 89 | 0.0 | 0.240 | 0.377 | 0.093 | 0.180 | 0.567 | 1.113 | 0.030 | 0.023 | 0.043 | 0.017 | 1.140 | 2.327 | 0.487 | 1.307 | 0.287 | 0.820 | 0.107 | 1.180 | nd | nd |
| 12/19/20 | 9.4 | 89 | 2.4 | 0.163 | 0.287 | 0.083 | 0.163 | 0.460 | 0.760 | 0.027 | 0.017 | 0.030 | 0.017 | 1.080 | 3.820 | 0.470 | 1.163 | 0.243 | 0.730 | 0.117 | 0.900 | nd | nd |
| 12/20/20 | 9.0 | 86 | 4.8 | 0.087 | 0.090 | 0.070 | 0.127 | 0.333 | 0.407 | 0.040 | 0.040 | 0.063 | 0.040 | 2.257 | 4.513 | 0.493 | 1.347 | 0.230 | 0.580 | 0.110 | 0.817 | nd | nd |
| 12/21/20 | 10.2 | 94 | 11.7 | 0.413 | 0.933 | 0.343 | 1.183 | 0.880 | 2.047 | 0.343 | 0.377 | 0.417 | 0.470 | 1.330 | 2.753 | 1.207 | 1.283 | 0.550 | 0.790 | 0.150 | 0.983 | nd | nd |
| 12/22/20 | 14.1 | 90 | 13.3 | 0.433 | 0.997 | 0.293 | 0.897 | 0.940 | 1.723 | 0.253 | 0.357 | 0.153 | 0.437 | 1.887 | 3.150 | 1.013 | 1.250 | 0.577 | 0.807 | 0.153 | 1.013 | nd | nd |
| 12/23/20 | 11.8 | 85 | 18.2 | 0.210 | 0.120 | 0.037 | 0.043 | 0.247 | 0.320 | 0.040 | 0.027 | 0.030 | 0.007 | 2.647 | 4.093 | 0.583 | 1.133 | 0.540 | 0.827 | 0.160 | 0.930 | nd | nd |
| 12/24/20 | 5.7 | 89 | 2.0 | nd | nd | nd | nd | nd | nd | nd | nd | nd | nd | nd | nd | nd | nd | nd | nd | nd | nd | nd | nd |
| 12/25/20 | 3.2 | 88 | 0.2 | nd | nd | nd | nd | nd | nd | nd | nd | nd | nd | nd | nd | nd | nd | nd | nd | nd | nd | nd | nd |
| 12/26/20 | 3.4 | 85 | 0.0 | 0.273 | 0.060 | 0.040 | 0.033 | 0.340 | 0.410 | 0.020 | 0.010 | 0.027 | 0.020 | 1.823 | 3.153 | 0.173 | 0.147 | 0.133 | 0.173 | 0.083 | 0.307 | nd | nd |
| 12/27/20 | 5.5 | 90 | 9.3 | 0.237 | 0.193 | 0.090 | 0.150 | 0.363 | 0.753 | 0.160 | 0.147 | 0.170 | 0.160 | 1.840 | 3.453 | 0.887 | 0.183 | 0.370 | 0.540 | 0.177 | 0.933 | nd | nd |
| 12/28/20 | 3.5 | 91 | 11.7 | 0.177 | 0.197 | 0.113 | 0.220 | 0.507 | 1.123 | 0.230 | 0.153 | 0.230 | 0.173 | 1.817 | 2.173 | 1.263 | 0.953 | 0.703 | 0.753 | 0.147 | 1.443 | nd | nd |
| 12/29/20 | 3.8 | 93 | 2.2 | 0.187 | 0.177 | 0.247 | 0.287 | 0.550 | 1.160 | 0.213 | 0.137 | 0.213 | 0.180 | 1.880 | 2.410 | 1.200 | 1.043 | 0.830 | 0.773 | 0.210 | 1.617 | nd | nd |
| 12/30/20 | 4.3 | 89 | 7.5 | 0.177 | 0.233 | 0.290 | 0.343 | 0.647 | 1.210 | 0.157 | 0.123 | 0.137 | 0.077 | 1.137 | 2.983 | 1.437 | 1.393 | 0.743 | 1.057 | 0.233 | 1.920 | nd | nd |
|  |  |  |  |  |  |  |  |  |  |  |  |  |  |  |  |  |  |  |  |  |  |  |  |
